# The CALLY index in cancer research: a comprehensive review

**DOI:** 10.3389/or.2026.1737805

**Published:** 2026-02-19

**Authors:** Yangzheng Zhang, Weidong Liu

**Affiliations:** Department of Gastrointestinal Surgery, The Affiliated Huaian No.1 People’s Hospital of Nanjing Medical University, Nanjing Medical University, Huai’an, Jiangsu, China

**Keywords:** biomarker, CALLY index, cancer, precision medicine, prognosis

## Abstract

This review focuses on the CALLY index in cancer, exploring its theoretical foundations, epidemiological insights, diagnostic and prognostic applications, therapeutic implications, controversies, and future perspectives. The CALLY index, based on C - reactive protein, albumin, and lymphocyte levels, has shown potential in predicting cancer prognosis and guiding treatment. Multiple studies across various cancer types indicate its association with survival outcomes, yet its widespread clinical adoption faces challenges. By synthesizing current research, this review aims to provide a comprehensive understanding of the CALLY index, highlighting its strengths, limitations, and potential for advancing cancer management in precision medicine.

## 1 Theoretical foundations of the CALLY index in cancer

### 1.1 Development and validation of the CALLY index

The CALLY index has shown prognostic potential in oncology. Emerging evidence, including a meta-analysis in digestive cancers and a recent study in oral squamous cell carcinoma (OSCC) where it independently predicted survival [Tsai et al. (1)], supports its utility in specific malignancies. This suggests it is a promising prognostic tool, particularly in cancers like OSCC and digestive cancers, though broader validation is needed (1).

In digestive system cancers, a meta - analysis of 18 articles (19 studies, 7,951 patients) demonstrated the prognostic value of the CALLY index (2). A lower CALLY index was significantly associated with poorer outcomes across all survival endpoints. The pooled HR for overall survival (OS) was 1.973 (95% CI: 1.734–2.244), with HRs for disease - free survival (DFS), recurrence - free survival (RFS), and cancer - specific survival (CSS) being 2.093 (95% CI: 1.682–2.604), 1.462 (95% CI: 1.292–1.654), and 2.456 (95% CI: 1.887–3.221), respectively (all P < 0.001). These findings validate the CALLY index as a reliable prognostic tool in different cancer contexts (Table 1).

**TABLE 1:** Prognostic value of the CALLY index in various cancers.

Cancer type	Study design (n)	Key findings related to CALLY index	Optimal cut-off	OS	DFS	RFS	CSS	Observations	Reference
Digestive system cancers									
Digestive system cancers (meta-analysis)	Meta-analysis (7,951)	Low CALLY significantly associated with poorer OS, DFS, RFS, and CSS. (pooled HR for OS: 1.97)	Varied	✓	✓	✓	✓	Analysis includes multiple cancer types within digestive system	(2)
Esophageal cancer	Retrospective (180)	Independent prognostic factor. Lower 5-year OS in low CALLY group (42.6% vs. 66.6%)	Not specified	✓	​	​	​	Patients who received curative treatment	(3)
Gastric cancer	Retrospective (617)	Independent predictor for OS and CSS. AUC was the highest among four biomarkers	Not specified	✓	​	​	✓	Calculated using formula: [Albumin × lymphocytes]/[CRP × 10^4]	(15)
Pancreatic cancer	Retrospective (461)	Preoperative CALLY index was an independent prognostic factor for OS and RFS.	1.9	✓	​	✓	​	Low CALLY associated with decreased OS (22.1 vs. 37.9 months) and RFS (12.4 vs. 16.4 months)	(14)
Distal cholangiocarcinoma	Retrospective (143)	Independent predictor of DFS and OS. Preoperative CALLY <3.5 associated with poorer outcomes	3.5	✓	✓	​	​	Patients underwent pancreaticoduodenectomy	(18)
Obstructive colorectal cancer (with stent)	Multicenter (263)	Low CALLY index was an independent predictor of postoperative complications (OR 1.961)	Not specified	​	​	​	​	Study focused on surgical complications, not survival endpoints	(7)
Gynecologic cancer									
Epithelial ovarian cancer	Cohort (310)	CALLY index ≥3 associated with better OS and DFS in both training and validation cohorts	3.0	✓	✓	​	​	FIGO stage, lymphatic metastasis, and CALLY index were prognostic factors	(4)
Thoracic cancers									
Non-small cell lung cancer	Cohort (INSCOC database)	Independent risk factor. CALLY-based nomogram showed good predictive power (C-index: 0.697)	Not specified	✓	​	​	​	Sex, TNM stage, surgical treatment, BMI, CALLY, and HGS were independent risk factors	(13)
Esophageal squamous cell carcinoma	Retrospective (318)	Low CALLY index associated with significantly worse 5-year CSS (21.8% vs. 62.6%)	Not specified	​	​	​	✓	Patients underwent radical excision; optimal threshold determined by restricted cubic spline model	(10)
Other solid tumors									
Breast cancer	Retrospective (174)	A novel prognostic biomarker. CALLY ≥2.285 associated with better survival outcomes	2.285	✓	✓	​	​	TNM stage and CALLY index were prognostic factors affecting OS and DFS.	(12)
Metastatic melanoma (on anti-PD-1)	Retrospective (92)	Independent predictor of OS. Low CALLY (≤2) linked to worse median OS (9.6 vs. 31.3 months)	2.0	✓	​	​	​	Patients treated with anti-PD-1 therapy	(16)
Hepatocellular carcinoma (undergoing TACE)	Retrospective	Low CALLY score associated with lower median OS (9.0 vs. 24.0 months)	Not specified	✓	​	​	​	Patients undergoing transarterial chemoembolization (TACE)	(19)
Non-metastatic nasopharyngeal cancer	Study (1,707)	CALLY-EBV DNA index served as an independent prognostic factor for OS, DMFS, and LRRFS.	Not specified	✓	​	​	​	Combined CALLY score with EBV DNA levels for risk stratification. (DMFS/LRRFS not mapped to DFS/RFS/CSS)	(11)
Population-based study									
NHANES (cancer patients)	Cohort (3,511)	Elevated CALLY index correlated with diminished risk of all-cause mortality (HR = 0.82 per ln unit)	Not specified	✓	​	​	​	Based on national health and nutritional examination surveys data	(8)

Abbreviations: OS, overall survival; DFS, disease-free survival; RFS, recurrence-free survival; CSS, cancer-specific survival; HR, hazard ratio; AUC, area under the curve; TACE, transarterial chemoembolization; EBV, Epstein-Barr virus; DMFS, distant metastasis-free survival; LRRFS, locoregional relapse-free survival; BMI, body mass index; HGS, handgrip strength; NHANES, national health and nutritional examination surveys; OR, odds ratio.

The checkmark (✓) indicates that the survival endpoint corresponding to the column was evaluated and reported in the relevant study.

### 1.2 Prognostic associations and biological rationale of the CALLY index in cancer

The CALLY index reflects the immune, nutritional, and inflammatory status of cancer patients, which are crucial aspects in cancer progression. In a study of 180 patients with esophageal cancer who received curative treatment, the 3 - and 5 - year overall survival rates were 50.0% and 42.6%, respectively, in the CALLY index - low group, and 75.9% and 66.6% in the CALLY index - high group (3). Univariate and multivariate analyses demonstrated that the CALLY index was an independent prognostic factor [hazard ratio = 2.310, 95% confidence interval = 1.416–3.767, p < 0.001]. This indicates that the underlying biological mechanisms related to inflammation, nutrition, and immunity, as represented by the CALLY index, significantly impact cancer prognosis.

In epithelial ovarian cancer, a study with 190 patients in the training cohort and 120 in the validation cohort found that the CALLY index ≥3 was associated with better survival outcomes in both cohorts (4). Univariate and multivariate COX analysis revealed that FIGO stage, lymphatic metastasis, and CALLY index were prognostic factors for both OS and DFS. The CALLY index likely influences cancer cell growth, metastasis, and the body’s immune response, although further research is needed to fully elucidate the detailed biological pathways involved.

### 1.3 Comparative analysis of prognostic indices in oncology

When comparing the CALLY index with other prognostic indices in oncology, several studies have evaluated different markers. For example, in a study on patients with resectable gastric and esophagogastric junction adenocarcinoma, neutrophil - to - lymphocyte ratio (NLR), platelet - to - lymphocyte ratio (PLR), lymphocyte - to - monocyte ratio (LMR), modified Glasgow prognostic score (mGPS), and prognostic nutritional index (PNI) were compared as preoperative indices (5). Preoperative NLR, PLR, LMR, and PNI were significantly associated with overall survival (OS) and relapse - free survival (RFS) in univariate Cox regression analysis. However, on multivariate Cox regression analysis, only preoperative PNI was independently associated with OS and RFS.

In metastatic colorectal cancer, various hematologic ratios and parameters such as the platelet - neutrophil to lymphocyte ratio (PNLR), PLR, NLR, Abnormal Hematological Markers Index (AHMI), and neutrophil - platelet score (NPS) were compared (6). Progression - Free Survival (PFS) hazard ratios (HR) between the high - risk and low - risk groups for PNLR was 2.0 (95% CI 1.28–3.13), for PLR was 1.74 (95% CI 1.13–2.67), for NLR was 1.54 (95% CI 1.04–2.29), for AHMI was 1.62 (95% CI 1.06–2.46), and for NPS was 1.47 (95% CI 1.1–1.96). It is important to clarify that while the studies cited above provide valuable context regarding the prognostic utility of various hematological indices, they do not directly compare these indices with the CALLY index. The CALLY index, by integrating markers of inflammation (CRP), nutrition (albumin), and immunity (lymphocytes) into a single score, represents a distinct composite approach. Future research that includes direct, head-to-head comparisons within the same patient cohorts is needed to rigorously position the CALLY index among these existing prognostic tools and to determine its unique or incremental value in cancer prognostication.

## 2 Epidemiological insights into the CALLY index and cancer survival

### 2.1 Population - based studies on the CALLY index in cancer patients

Studies have investigated the CALLY index in cancer patients across different settings and cancer types. In a Japanese multicenter study of 263 patients with obstructive colorectal cancer managed with a colonic stent, patients were classified into a low CALLY index group (n = 85) and a high CALLY index group (n = 178) (7). The CALLY - L group had greater blood loss (53 mL vs. 20 mL, p = 0.002), higher poor performance status (PS3; 20% vs. 10.1%, p = 0.033), open surgery (21.2% vs. 7.3%, p = 0.001), distant metastases (41.2% vs. 20.8%, p = 0.01), and postoperative complications (30.6% vs. 18.5%, p = 0.039) than the CALLY - H group. Multivariate analysis identified a low CALLY index as an independent predictor of complications (odds ratio 1.961, 95% confidence interval 1.013–3.795; p = 0.045). This study, although multi-institutional, demonstrates the utility of the CALLY index in predicting surgical outcomes in a specific clinical scenario.

Larger-scale analyses provide broader insights. A study using data from the National Health and Nutritional Examination Surveys (NHANES) on 3511 cancer - afflicted adults found that an elevated CALLY index correlated with a diminished risk of all - cause mortality (8). Over a median follow - up of 103 months, 1,355 deaths occurred. Applying a natural logarithmic transformation to the CALLY index, the comprehensively adjusted model revealed that each one - unit increment in ln CALLY corresponded to an 18% decrease in all - cause mortality risk among cancer patients (HR = 0.82, 95% CI:0.79–0.86). These findings from a large, nationally representative cohort underscore the potential of the CALLY index as a general prognostic marker in cancer patients.

### 2.2 Demographic variations in the CALLY index impact

Research on demographic variations in the impact of the CALLY index has been limited but provides some insights. A study examining the correlation between the CALLY index and sarcopenia risk used two cohorts: 1804 community dwellers from the NHANES database in the United States and 139 patients from the Department of Gerontology at Kunshan Hospital, China (9). Results demonstrated a significant non - linear relationship between the CALLY index and the risk of sarcopenia (P < 0.001). Elevated levels of the CALLY index were independently linked to a decreased risk of sarcopenia in both community residents (OR = 0.35, 95% CI 0.20–0.57, Q3 CALLY index and OR = 0.26, 95% CI 0.11–0.56, Q4 CALLY index) and hospitalized patients (OR = 0.35, 95% CI 0.12–0.96). While not directly focused on cancer, this study suggests that the association between a higher CALLY index and better health outcomes may be consistent across different populations (U.S. community residents and Chinese hospitalized patients). However, further research is needed to specifically explore how age, gender, ethnicity, and other demographic factors interact with the CALLY index in the context of cancer prognosis.

### 2.3 Longitudinal trends in CALLY index research

Longitudinal trends in CALLY index research show an increasing interest in its prognostic value. A meta - analysis on digestive system cancers, which included 18 articles (19 studies, 7,951 patients), demonstrated the consistent prognostic significance of the CALLY index over different treatment strategies, cancer types, cutoff values, sample sizes, and regions (2). A lower CALLY index was significantly associated with poorer outcomes across all survival endpoints. In a study on esophageal squamous cell carcinoma, the prognostic value of the CALLY index was validated in 318 patients who underwent radical excision (10). The 5 - year cancer - specific survival (CSS) was significantly worse in patients with a low CALLY index (21.8% vs. 62.6%, P < 0.001). These studies, along with others, contribute to the growing body of evidence supporting the CALLY index as a reliable prognostic tool over time. Future longitudinal research could further explore how the CALLY index changes in relation to disease progression, treatment response, and long - term survival in different cancer patient populations.

## 3 Diagnostic and prognostic applications of the CALLY index in cancer

### 3.1 Prognostic value of the CALLY index in different cancer types

The CALLY index has shown prognostic value across various cancer types. In non - small cell lung cancer, a study of patients followed up in the INSCOC database found that sex, TNM stage, surgical treatment, BMI, CALLY, and HGS were independent risk factors for prognosis (11). The OS of NSCLC patients with a low CALLY index score was significantly worse than that of patients with a high CALLY index (P < 0.001). The CALLY - based nomogram had a good predictive prognostic power, with a C - index of 0.697.

In breast cancer, a study of 174 patients showed that the CALLY index was a new prognostic biomarker (12). The cut - off value of the CALLY index was determined at 2.285. Patients with CALLY ≥2.285 had better survival outcomes. Multivariate Cox analysis showed that TNM stage and CALLY index were prognostic factors affecting OS and DFS. These results across different cancer types highlight the broad applicability of the CALLY index in predicting cancer prognosis.

### 3.2 Integration of the CALLY index in cancer diagnostic protocols

The CALLY index has the potential to be integrated into cancer prognostic and risk-stratification protocols. In a study of 1707 non - metastatic nasopharyngeal cancer patients, the CALLY score was combined with Epstein - Barr virus (EBV) DNA levels to develop a comprehensive index (13). By integrating these factors, patients were categorized into three risk clusters, and significant differences in overall survival (OS), distant metastasis - free survival (DMFS), and locoregional relapse - free survival (LRRFS) were observed among different risk groups (all P values <0.05). Multivariate analysis revealed that the CALLY - EBV DNA index served as an independent prognostic factor for these survival endpoints. This suggests that the CALLY index, when combined with other relevant markers, could enhance the accuracy of patient risk stratification and prognosis assessment.

In pancreatic cancer, a study of 461 patients who underwent surgical resection found that the preoperative CALLY index was an independent prognostic factor for overall survival (OS) and relapse - free survival (RFS) (14). The optimal cut - off value for the preoperative CALLY index was 1.9. A low CALLY index was associated with decreased OS (22.1 vs. 37.9 months) and RFS (12.4 vs. 16.4 months). These findings indicate that the CALLY index could be incorporated into prognostic algorithms to predict patient outcomes and guide treatment decisions in pancreatic cancer.

### 3.3 Clinical case studies: CALLY index in surgical oncology

Clinical case studies in surgical oncology further illustrate the utility of the CALLY index for prognostication. In a study of 617 gastric cancer patients who underwent gastrectomy, the CALLY index was calculated using the formula [albumin (g/dL) × lymphocytes (/μl)]/[CRP (mg/dL) × 104] (15). Receiver operating characteristic analysis showed that the area under the curve for the CALLY index was the highest among four biomarkers. The 5 - year overall survival (OS) and cancer - specific survival (CSS) rates in the low and high CALLY groups were statistically significant. Multivariate analysis identified the CALLY index as an independent factor for OS and CSS.

In esophageal squamous cell carcinoma, 318 patients who underwent radical excision were studied (10). A restricted cubic spline (RCS) model was used to determine the optimal threshold of CALLY. Patients with a low CALLY index experienced significantly worse 5 - year CSS (21.8% vs. 62.6%, P < 0.001). These case studies demonstrate the practical significance of the CALLY index in predicting patient outcomes in real - world surgical oncology settings.

## 4 Therapeutic implications of the CALLY index in cancer management

### 4.1 Personalized treatment strategies based on the CALLY index

The CALLY index can potentially guide personalized treatment strategies in cancer. In metastatic melanoma patients treated with anti - PD - 1 therapy, a retrospective study of 92 patients found that the optimal CALLY index cutoff was 2 (16). Patients with a low CALLY index (≤2) had worse median overall survival (OS) and progression - free survival (PFS) compared to those with a high CALLY index (>2) (median OS: 9.6 vs. 31.3 months, p < 0.001; median PFS: 3.8 vs. 10.6 months, p = 0.001). Multivariate analysis identified the CALLY index as an independent predictor of OS. This suggests that patients with a low CALLY index, indicative of a poorer prognosis, might be candidates for more intensive monitoring, combination therapies, or enrollment in clinical trials testing novel strategies to overcome potential resistance mechanisms. The index could thus inform risk-adapted therapeutic approaches.

In epithelial ovarian cancer, the CALLY index was found to be a prognostic factor for patients undergoing surgery (4). With a cut - off value of 3, patients with a CALLY index ≥3 had better survival outcomes. This knowledge could assist in personalizing postoperative management. For instance, patients with a high CALLY index (better prognosis) might be optimally managed with standard adjuvant therapy, while those with a low index (worse prognosis) could be discussed for more aggressive or extended adjuvant regimens, where the potential survival benefit is weighed against increased toxicity. Tailoring treatment intensity based on the patient’s CALLY index-related prognosis represents a step towards personalized care.

### 4.2 Impact of the CALLY index on treatment outcomes

The CALLY index has a significant impact on treatment outcomes in cancer patients. In a study of 826 gastric cancer patients who underwent gastrectomy, the cut - off of the CALLY index was 2 (17). The 147 patients with a preoperative CALLY index <2 had significantly worse overall survival (OS) and relapse - free survival (RFS) than those with a CALLY index ≥2 (P < 0.01 for both). Multivariate analysis identified a CALLY index <2 as an independent predictor of worse RFS and OS. This indicates that patients with a low CALLY index may require more intensive treatment strategies to improve their outcomes.

In distal cholangiocarcinoma, a study of 143 patients who underwent pancreaticoduodenectomy found that the CALLY index was an independent predictor of disease - free survival and overall survival (18). Eighty - seven (61%) patients had a preoperative CALLY index <3.5. In multivariate analysis, obstructive jaundice drainage, poorly differentiated tumor, and CALLY index <3.5 were independent predictors of both survival endpoints. These results suggest that the CALLY index can help in predicting treatment response and tailoring treatment plans to improve patient outcomes.

### 4.3 Potential role of the CALLY index in monitoring therapy response

The CALLY index has been studied for its potential to reflect therapy response. While current evidence robustly supports its prognostic value, its utility for dynamic monitoring of treatment efficacy requires further validation. In a study of patients with hepatocellular carcinoma undergoing transarterial chemoembolization (TACE), a low CALLY score was associated with low median overall survival (OS) (low vs. high CALLY: 9.0 vs. 24.0 months, p < 0.001) (19). In the multivariate analysis, the CALLY index remained an independent prognostic predictor (p = 0.008). These findings establish its baseline prognostic value. It is hypothesized that changes in the CALLY index during or after TACE could potentially reflect the effectiveness of the treatment, but this specific application needs prospective investigation.

In summary, the CALLY index has demonstrated consistent baseline prognostic value across various cancers, which provides a theoretical foundation for its further evaluation in dynamic treatment monitoring. However, establishing it as a routine tool for monitoring therapy requires future studies to directly validate the association between its serial changes and radiological, pathological response, or survival benefits during the course of treatment.

## 5 Controversies and challenges in CALLY index research

### 5.1 Limitations of the CALLY index in cancer prognostication

Despite its promise, the CALLY index has limitations in cancer prognostication. While it reflects the immune, nutritional, and inflammatory status, it may not capture all aspects of cancer biology. In some studies, although the CALLY index was significantly associated with survival outcomes, the concordance index (C - index) values, which measure the discriminatory ability of a prognostic model, were relatively modest. For example, in a study on hepatocellular carcinoma patients undergoing TACE, the C - index of the CALLY index was 0.60, similar to other established immunonutritive and inflammation scoring systems (range: 0.54–0.63) (19). This indicates that the CALLY index may not be highly discriminatory in all cases.

Also, the cut - off values for the CALLY index vary across different studies and cancer types. In colorectal cancer, the cutoff value was determined to be 2 in one study (20), while in pancreatic cancer, it was 1.9 (14). This lack of a standardized cut - off value makes it difficult to compare results across studies and may limit its widespread adoption in clinical practice.

### 5.2 Debates on the clinical utility of the CALLY index

There are debates on the clinical utility of the CALLY index. Some researchers question its incremental value over existing prognostic markers. In a study comparing the CALLY index with other biomarkers in gastric cancer, while the CALLY index was an independent predictor of OS and CSS, it was not clear whether it provided additional information beyond traditional clinicopathological factors (15). The modified Glasgow prognostic score (mGPS) and neutrophil - lymphocyte ratio (NLR) also showed prognostic significance, and it was debated whether the CALLY index offered unique advantages in clinical decision - making.

Another aspect of the debate is related to the complexity of incorporating the CALLY index into routine clinical practice. Since it requires the measurement of C - reactive protein, albumin, and lymphocyte levels, there may be concerns about the cost - effectiveness, especially in resource - limited settings. Additionally, the need for accurate and standardized laboratory measurements to calculate the CALLY index may pose challenges in some healthcare facilities.

## 6 Future perspectives on the CALLY index in cancer

### 6.1 Standardization of methodology and integration with novel analyses

The future utility of the CALLY index depends on methodological standardization and innovation. Firstly, efforts should be made to standardize the calculation and cut - off values of the CALLY index across different cancer types. This would enable more accurate comparison of results between studies and facilitate its clinical implementation. Secondly, research could explore the combination of the CALLY index with other emerging biomarkers or molecular signatures. For example, in nasopharyngeal cancer, combining the CALLY index with EBV - DNA levels showed enhanced prognostic performance (13). Similar combinations in other cancer types could potentially improve the accuracy of prognostication. Furthermore, prospective studies are required to validate its role in guiding treatment decisions and predicting response, supported by long-term follow-up and deeper investigation into its biological mechanisms. Additionally, innovation in assays for measuring CRP, albumin, and lymphocytes—including non-invasive methods—could improve precision and clinical feasibility.

Innovations in CALLY index methodologies could further enhance its utility. Machine learning (ML) techniques could be applied to better analyze the relationship between the CALLY index and cancer outcomes. In a study on acute myocardial infarction, the Extreme Gradient Boosting (XGBoost) ML algorithm was used to predict the no - reflow phenomenon using the CALLY index (21). The model obtained with the XGBoost algorithm showed high accuracy rates in predicting no - reflow status, demonstrating the potential of ML in leveraging the CALLY index for more accurate predictions. Similar approaches could be applied in cancer research to identify complex patterns and interactions between the CALLY index and other clinical variables.

### 6.2 Role in therapeutic stratification and emerging treatment contexts

The CALLY index has potential in emerging cancer therapies. In immunotherapy, such as in metastatic melanoma patients treated with anti - PD - 1 therapy, the CALLY index was found to be an independent prognostic biomarker (16). It could potentially be used to identify patients who are more likely to benefit from immunotherapy, helping to optimize treatment selection. As new immunotherapies and targeted therapies are developed, the CALLY index may play a role in predicting patient response.

In combination therapies, the CALLY index could help in tailoring the treatment regimen. For example, in digestive system cancers, understanding how the CALLY index relates to the response to chemotherapy or targeted agents could guide the selection of the most appropriate combination. By stratifying patients based on their CALLY index, clinicians could potentially improve treatment outcomes and reduce unnecessary toxicity. A pivotal future application to be studied is whether such stratification can aid in decisions to forego potentially futile, high-toxicity treatments in patients with a very poor prognostic profile.

### 6.3 Vision for integration into a precision oncology framework

The integration of the CALLY index in precision medicine holds great promise. In precision medicine, the goal is to provide personalized treatment based on an individual patient’s characteristics. The CALLY index, which reflects inflammation, nutrition, and immunity, can contribute to this by helping to stratify patients into different risk groups. In esophageal squamous cell carcinoma, the CALLY index, when combined with TNM stage, could create a new staging system that outperformed the traditional TNM classification for prognostication (10).

In the context of lung cancer, the integrative analysis of omic, clinical, and epidemiological data is a core principle of precision medicine (22). The CALLY index could be incorporated into this framework, along with other biomarkers, to provide a more comprehensive understanding of the patient’s disease state. This could lead to more accurate prediction of prognosis, selection of the most appropriate treatment, and ultimately, improved patient outcomes in the era of precision medicine.
